# First initiative to develop a standard methodology for the evaluation of Attractive Targeted Sugar Baits in different settings against targeted mosquito vectors: a methodological review

**DOI:** 10.1186/s12936-025-05545-w

**Published:** 2025-10-09

**Authors:** Appadurai Daniel Reegan, Sam Joy, Purushotham Jambulingam, Manju Rahi

**Affiliations:** 1 ICMR-Vector Control Research Centre, Indira Nagar, Puducherry, 605 006 India; 2 Academy of Scientific and Innovative Research (AcSIR), CSIR-Human Resource Development Centre, (CSIR-HRDC) Campus, Ghaziabad, Uttar Pradesh 201 002 India; 3 Indian Council of Medical Research, New Delhi, 110 029 India

**Keywords:** ATSB, Protocol, Vector control tool, Methodology

## Abstract

**Background:**

Vector-borne diseases remain a major global health problem, mostly in tropical and subtropical areas. Effective vector control is crucial for controlling vector borne diseases (VBDs). Over the years various vector control tools and strategies have been employed globally. However, the recent challenges including insecticide-resistant, alterations in vector behaviour, and non-target effects have highlighted the need for novel vector control tools and alternate strategies. One such tool is the Attractive Targeted Sugar Baits (ATSBs), which uses the sugar-seeking habit of adult mosquitoes. The ATSB strategy operates on an “attract and kill” approach, where mosquitoes are lured to the bait and to feed on sugar combined with an insecticide. For this, a standard methodology needs to be developed for a uniform evaluation of ATSBs.

**Results:**

The ATSB vector control strategy has shown promising results in studies carried out in various parts of Africa and the Middle East on controlling populations of mosquito species. Although numerous experiments have been conducted and are ongoing in various countries, there remains a lack of standardized guidelines for evaluating ATSBs. In 2023, the ICMR along with partners drafted the 3rd edition of Common Protocols for evaluating public health vector control products. The revised edition included a trial methodology for ATSB. Taking this into consideration, the phase-wise standard methodology is presented in this review for the uniform evaluation of different formulations/products of ATSBs.

**Conclusions:**

The methodologies, outlined in this article will serve as the standard methodology for testing ATSB formulations/products under laboratory conditions (Phase I), small-phase (Phase II), and large-phase field trial (Phase III) conditions.

## Background

Vector-borne diseases (VBDs) contribute considerably to the worldwide disease burden for about 17% of all communicable diseases. These diseases result in more than 700,000 annual deaths, with the burden specifically high in tropical and subtropical countries [1]. Vector-based interventions are an essential component in the control and elimination of VBDs. The Integrated Vector Management (IVM) strategy, employing a variety of vector control products, tools, and strategies, has been in use for the past few decades and has made considerable outcome to the control and prevention of VBDs. These vector control tools/products utilize various strategies to reduce the population of disease-carrying vectors, thereby preventing the transmission of VBDs. Some of these key tools and strategies include long-lasting insecticidal nets, space sprays, indoor residual spray, chemical and biological-based larvicide, environmental modification actions such as breeding habitat reduction, and genetic modifications of vectors. The increasing challenges in recent years posed by factors viz global environmental changes (climate changes), insecticide-resistance of vectors, alterations in vector behaviour, vector expansion into new regions, and concerns regarding non-target effects and environmental safety have directly impacted the vector control strategies in several ways and thereby highlights the need for novel vector control tools and alternate strategies towards control and prevention of VBDs [2]. Development of these novel/alternative vector control tools requires thorough scientific evaluation through laboratory and field trials, with documented evidence of their impact on vector disease control, before implementing as public health tool. Some of the promising novel vector control tools include products such as synergist-treated insecticide nets, endectocides, novel synthetic pyrethroids, neonicotinoids, non-pyrethroid compounds, genetically modified vectors, newer formulations for indoor residual spray (IRS), newer strains for use in larvicides, and insecticide-treated wearables [68].

### Attractive Targeted Sugar Baits (ATSBs)

ATSB signifies an innovative class of vector management that leverages the benefit of the sugar-seeking behaviour of adult mosquitoes to kill both male and female mosquitoes [3]. In this method, any fruit juice or flower aroma that is attractive is used as an attracting agent, sugar infusion as a luring food, and any chemical insecticide that is toxic is used to kill adult vector mosquitoes. Various researchers have reported to have studied different ATSBs with different insecticides and formulations [4–7]. Various mosquitocides such as boric acid, spinosad, eugenol, pyriproxyfen, dinotefuron, ivermectin, and micro-coated garlic oil have been tested in both laboratory and field settings for ATSBs [8–10, 63]. ATSBs have been reported to be successful in controlling mosquito species both outdoors and indoors [8]. ATSBs has been assessed against *Anopheles* and *Culex* and *Phlebotomus* vectors in some countries either as a standalone tool for vector control or as an additional intervention tool to other vector control strategies. Table 1 and Table 2 summarizes various studies carried out on different formulations and bait stations of ATSBs conducted in laboratory, semi-field, and field conditions. A literature survey showed that fruit juices and sucrose solution were used in different percentages with insecticide to prepare ATSB. Many of the laboratory experiments recorded above 80% mortality and a few with 100% mortality (Table 1). Similarly, researchers have used different concentrations in the semi-field and field studies and reported varied activities (Table 2).

**Table 1:** Studies on Attractive Targeted Sugar Baits (ATSBs) in laboratory conditions

Sl. no	Place of study	Target vector species	Type of laboratory study (assay in cage cotton balls/filter papers/sprayed on cage plants)	Attractant used	Insecticides used	Concentration used	Results	Reference
1	Georgia	*Aedes aegypti*	Cage filter papers	20% sucrose solutions	Malathion	0.1, 0.25 0.5 and 1 ppm	60–100% mortality of the total mosquitoes exposed	Lea et al., [18]
2	USA	*Aedes albopictus* and *Anopheles quadrimaculatus, Culex Nigripalpus*	Cage cotton balls	10% sucrose solution	Boric acid	0.0001, 0.001, 0.01, 0.1 and 1%	≥ 98% mortality of the total mosquitoes exposed	Xue et al., [19]
3	USA	*Aedes albopictus*	Cage cotton balls	10% sucrose solution	Boric acid	0.1%	> 90% mortality of the total mosquitoes exposed	Ali et al., [20]
4	USA	*Culex quinquefasciatus*, *Anopheles quadrimaculatus* and *Aedes taeniorhynchus*	Cage cotton balls	10% sucrose	Bifenthrin, Cyfluthrin, Deltamethrin, Permethrin, Fipronil, Chlorfenapyr, Imidacloprid, Thiamethoxam, Spinosad and Ivermectin	Unknown	*Cx. quinquefasciatus* were most susceptible to fipronil with KD_50_ of 0.1 and *An. quadrimaculatus* and *Ae. taeniorhynchus* were most susceptible to imidacloprid with KD_50_ of 0.03 and 0.06, respectively	Allan et al., [6]
5	USA	*Aedes albopictus*	Cage cotton balls	5% sugar solution	Boric acid	1%	> 98% mortality of the total mosquitoes exposed	Xue et al., [21]
6	USA	*Aedes albopictus*	Cage cotton balls	29% Goya Mango juice and 29% Goya Guava juice and 21% brown sugar	Boric acid	1%	100% mortality of the total mosquitoes exposed	Naranjo et al., [22]
7	Morocco	*Aedes aegypti *and *Culex quinquefasciatus*	Cage cotton balls	Cactus bearing ripe fruit and10% sucrose	Dinotefuran	0.1, 1, 10, 100 and 1000 ppm	> 80% mortality of the total mosquitoes exposed	Khallaayoune et al., [23]
8	USA	*Aedes aegypti, Anopheles quadrimaculatus* and *Culex quinquefasciatus*	Cage cotton balls	10% sugar bait concentrate	Eugenol	0.1%, 1.0%, and 10%	100% mortality with 10%, 98% with 1%, and 97.3% with 0.1% of total *Ae. aegypti* exposed after 24 h 46.2% mortality with 10%, 87.5% with 1%, and 38.7% with 0.1% after 24 h for *An. Quadrimaculatus* 100% mortality with 10% and 0.1%, 98% with 1% of total *Cx. quinquefasciatus* after 24 h	Qualls et al., [24]
9	USA	*Aedes albopictus*	Cage cotton balls	5% sugar	Boric acid or eugenol and pyriproxyfen	1% and 1 mg/liter	63.3 ± 16% mortality of the total mosquitoes exposed with boric acid sugar baits with pyriproxyfen and 80.4 ± 3.3% mortality of the total mosquitoes exposed with eugenol sugar baits with pyriproxyfen	Fulcher et al., [25]
10	USA	*Aedes taeniorhynchus*	Cage cotton balls	5% sucrose	Boric acid	1%	52% mortality of the total mosquitoes exposed in black mangrove and 68% mortality of the total mosquitoes exposed in yaupon holly experiment	Hossain et al., [26]
11	USA	*Aedes albopictus*	Cage cotton balls	Mango chunks, lime juice and10% sucrose solution	Pyriproxyfen	0.5, 1, 5, and 10 ppb	Higher % emergence inhibition was achieved through fecal dissemination of 10 ppb (57%) of pyriproxyfen in ASB than 1 ppb and 0.5 ppb	Scott et al., [27]
12	USA	*Aedes albopictus*	Cage cotton balls	10% sucrose	Boric acid	1%	91.3% mortality of the total mosquitoes exposed	Wang et al., [28]
13	Australia	*Aedes aegypti*	Cage cotton balls	0.2 L each of guava nectar, mango nectar and water with 200 g brown sugar	Fipronil	0.06%	100% mortality of the total mosquitoes exposed	Fikrig et al., [29]
14	USA	*Aedes albopictus*	Cage cotton balls	Mango, Lime juice and sugar solution	Boric acid and Red food-grade dye	1% and 0.5%	The application of ATSB to various plant types resulted in significant mortality of adult mosquitoes after exposed to all treated plants, compared to untreated control plants	Seeger et al., [30]
15	Brazil	*Aedes aegypti*	Cage cotton balls	78% fruit juice (guava, mango and cupuaçu), brown sugar (15%)	Green food dye and boric acid	3% and 4%	81% mortality for male and 61% mortality for female of the total mosquitoes exposed	Barbosa et al., [31]
16	USA	*Aedes aegypti *and *Anopheles stephensi,*	Cage cotton balls	10% sucrose	Sodium ascorbate	6%, 8%, 10% and 20%	100% mortality of the total mosquitoes exposed with 20% ATSB for both *Ae. aegypti An. stephensi*	McDermott et al., [32]
17	Malaysia	*Aedes albopictus*	Cage cotton balls	0.1%, 0.5% and 1% sucrose solution	Boric acid	0.1%, 0.5% and 1%	Mortality of 12.4% after 24 h and 62.6% after 48 h with 1% boric acid ATSB exposure to total released mosquitoes	Abdullah et al., [33]
18	USA	*Aedes albopictus*	Cage cotton balls	Microencapsulated cin-namon oil-sesame oil	Yellow food dye and green food dye	0.5%	Mortality at 10% and 1% ATSB concentration was 95.8% and 90.0% mortality of the total mosquitoes, respectively, after 48 h	Traore et al., [34]
19	USA	*Aedes aegypti*	Cage cotton balls	10% sugar solution	Boric acid and Red food dye	0.5% and 0.25%	Sugar meals composed of arabinose, lactose, or cellobiose significantly reduced the survival of *Ae. aegypti* compared to sucrose controls	Airs et al., [35]
20	USA	*Aedes albopictus*	Cage cotton balls	0.18% yeast and 88.34% sucrose	Sodium chloride	11.48%	80% mortality of males and 30% mortality of females of the total mosquitoes	Aryaprema, [36]
21	Ecuador	*Aedes aegypti*	Cage cotton balls	10% sugar solution	Boric acid	1%	100% mortality of the total mosquitoes exposed	Sippy et al., [37]
22	USA	*Aedes aegypti*	Cage cotton balls	5% sucrose solution	Boric acid	1%	The average mortality of TSB-treated female mosquitoes was 90.8% ± 11.5 for PR and 99.3% ± 0.6 for ORL. Similarly, the TSB-treated males resulted in 96.9% ± 4.9 and 99.6% ± 0.6 mortality of total mosquitoes exposed for PR and ORL, respectively	Pearson et al., [38]
23	Bangladesh	*Aedes aegypti*	Cage cotton balls	10% sugar solution	Boric acid	0.5%, 0.75%, 1% and 2%	The highest mortality was found at 2% dose, with 96.67 ± 3.33 and 93.33 ± 1.67% for males and females of the total mosquitoes exposed, respectively	Hossain et al., [39]
24	Virginia	*Ae. j. japonicus*	Cage cotton balls	Mango, peach, blueberry, blackberry, grape juice and a cola soda	Boric acid	1%	100% mortality of exposed mosquitoes	Fryzlewicz et al., [40]
25	Mali	*Aedes aegypti, Aedes Albopictus* and *Culex quinquefasciatus*	Cage cotton balls	10% sucrose solution	Red and green food dyes, Boric acid and Bti	1% and 8%	97% mortality for *Ae. aegypti,* 98% mortality for *Ae. albopictus* and 100% mortality for *Cx. quinquefasciatus* of the total mosquitoes exposed	Davis et al., [41]
26	Brazil	*Aedes aegypti*	Cage cotton balls	Guava fruit and 10% brown sugar	A red food coloring	Unknown	The fecundity and fertility of the females were reduced by up to 51% and 97%, respectively	Silva et al., [42]
27	USA	*Aedes albopictus*	Cage cotton balls	10% sugar solution	Spinosad	1%	Ingestion of TSB caused a reduction in survival of females, but increased mosquito susceptibility to DENV infection, disseminated infection, and transmission	Alomar et al. [7]
28	India	*Aedes aegypti*, *Anopheles stephensi *and *Anopheles culicifacies*	Cage cotton pads and sprayed on cage plants	10% glucose solution	Boric acid	1%, 2%, 3% and 4%	100% mortality of exposed mosquitoes with 4% boric acid solution on *Ae. aegypti*, *An. stephensi* and 3% boric acid solution on *An. Culicifacies* 88.9—94.4% mortality in *An. stephensi* and *An. culicifacies* with 2% boric acid sprayed on plants	Kumar et al., [9]
29	USA	*Aedes aegypti* and *Anopheles stephensi*	Cage cotton balls	Citral, geraniol, thujone, linalool, ben-zaldehyde, anisaldehyde, gallic acid, 10% sucrose and 10% fructose	Sodium ascorbate	20%	Citral, Linalool, Anisaldehyde and Geraniol showed increased mortality of *Ae. Aegypti* Thujone caused the highest activation rate of 71.3 ± 9.28%. The addition of anisaldehyde significantly increased *An. stephensi* feeding rates	Tucker et al., [43]
30	India	*Aedes aegypti*	Cage cotton discs	Fruit juice and 10% sucrose solution	Deltamethrin/dinotefuran	Unknown	85–95% mortality of total mosquitoes exposed	Kumar et al., [44]
31	Tanzania	*Aedes aegypti*	Cage cotton balls	10% sugar solution	Ivermectin	0.005%, 0.01%, 0.015%, 0.02%, 0.025%, 0.03%, 0.04% and 0.05%	80% mortality of the total exposed mosquitoes within 24 h and > 90% within 48 h	Tenywa et al., [45]
32	Brasil	*Aedes aegypti*	Cage cotton balls	10, 50 and 70% sucrose solution	Ivermectin and red food coloring	100 ppm and 3%	80% mortality of the total mosquitoes exposed	Dias et al., [46]
33	Taiwan	*Aedes albopictus*	Cage cotton balls	10% sugar solution	Boric acid	1%	The mortality of female mosquitoes ranged from 7.8% to 16.7% but male mosquitoes recorded 37.8%–64.3% of the total mosquitoes exposed	Chiu et al., [47]
34	India	*Aedes aegypti*	Cage cotton discs	Guava and 10% sucrose solution	Deltamethrin	0.003125–0.8 mg/10 mL	8.33–97.44% mortality of the total mosquitoes exposed on AND-*Ae. aegypti* strain and 5.15–96.91% mortality on AND-*Ae. aegypti*-DL10 strain, while GVD-Delhi strain registered 2.04–95.83% mortality and SHD-Delhi strain showed 5.10–97.96% mortality	Kumar et al., [48]

**Table 2:** Studies on Attractive Targeted Sugar Baits (ATSBs) at small-scale and large-scale trials

Sl. no	Place of study	Target vector species	Type of study	Attractant used	Insecticides used	Concentration used	Results	Reference
1	Israel	*Anopheles sergentii*	Large-scale trial (outdoor) sprayed on plants	20% sucrose solution	Dye and spinosad	2% and 0.04%	∼ 100% mortality	Muller et al., [15]
2	USA	*Aedes albopictus, Culex nigripalpus,* and *Ochlerotatus taeniorhynchus*	Small-scale trial (outdoor)	5% sucrose solution	Boric acid	1%	80–100% mortality	Xue et al., [49]
3	Israel	*Anopheles claviger*	Large-scale trial (outdoor) as ATSB traps	85% nectarines, 15% brown sugar	Red food dye and *spinosad*	0.5% and 0.04%	~ 90% reduction in mosquito density after 4 weeks	Muller and Schlein, [50]
4	Israel	*Anopheles sergentii *and *Aedes caspius*	Large-scale trial (outdoor)	75% juice of nectarines, 5% red wine and 10% brown sugar	Red food dye, Spinosad and BaitStab™	0.5%, 0.04% and 10%	91% mortality for *An. sergentii* and 67% mortality for *Ae Caspius*	Muller et al., [4]
5	USA	*Aedes aegypti *and *Ochlerotatus taeniorhynchus*	Small-scale trial and large-scale trial (outdoor)	10% sucrose solution	Boric acid and fipronil	1% and 0.1%	60–65% reduction in landing rate after 48 h and 32% reduction in landing rate after 96 h	Xue et al., [51]
6	Mali	*Anopheles gambiae *and *Anopheles arabiensis*	Large-scale trial (outdoor)	30% Guava juice, 30% Honey Melon juice, 25% water, 12% brown Sugar, 2% local millet beer	Boric acid and BaitStab™	1%	Reduction by ∼ 90% after 1 week	Muller et al., [5]
7	Mali	*Anopheles gambiae*	Large-scale trial (outdoor)	Fruit juice and seedpods	Unknown	Unknown	Guava and honey melon were the two most attractive fruits	Müller et al., [52]
8	USA	*Aedes albopictus*	Small-scale trial (outdoor)	5% sugar solution	Boric acid	1%	> 93% mortality	Xue et al., [22]
9	Israel	*Anopheles sergentii*	Large-scale trial (outdoor) sprayed on plants	∼75% juice of pear cactus, 5% wine, 20% brown sugar	Boric acid and BaitStab™	1%	> 95% mortality	Beier et al., [53]
10	USA	*Aedes albopictus*, *Anopheles crucians*, *Culex quinquefasciatus*, and *Toxorhynchites rutilus rutilus*	Large-scale trial (outdoor)	95% overripe/rotting Plums, 5% red win and 10% brown sugar	Orange food dye and BaitStab™	0.5% and 10%	95% of the mosquitoes trapped were stained	Qualls et al., [54]
11	Morocco	*Culex quinquefasciatus, Aedes aegypti *and *Aedes caspius*	Large-scale trial (outdoor) sprayed on plants	Cactus bearing ripe fruit and10% sucrose	Dinotefuran	0.1, 1, 10, 100 and 1000 ppm	> 70% reduction of mosquito populations after 3 weeks	Khallaayoune et al., [24]
12	USA	*Aedes albopictus*	Small-scale trial and large-scale trial (Outdoor)	29% Goya Mango juice and 29% Goya Guava juice and 21% brown sugar	Boric acid	1%	95% mortality 58% reduction after 7 days	Naranjo et al., [23]
13	Tanzania	*Anopheles arabiensis, Anopheles gambiae s.s *and *Culex quinquefasciatus*	Large-scale trial (indoor) as ATSB trap	35% guava juice, 10% brown sugar	Red food dye, boric acid, chlorfenapyr and tolfenpyrad	2%, 2%, 0.5% and 1%	Chlorfenapyr, boric acid and tolfenpyrad ATSB killed 100%, 85% and 86% of *An. gambiae* s.s. and 48%, 41% and 45% of *An. arabiensis* within 24 h	Stewart et al., [55]
14	USA	*Aedes taeniorhynchus*	Large-scale trial (outdoor)	5% sucrose	Boric acid	1%	∼40% reduction in the field after 48 h	Hossain et al., [27]
15	USA	*Aedes atlanticus, Aedes. infirmatus, Anopheles crucians, Culex nigripalpus, Culiseta melanura, Culex erraticus* and *Uranotaenia sapphirina*	Large-scale trial (outdoor) sprayed on plants	10% sugar bait concentrate	Eugenol	0.1%, 1.0%, or 10%	> 70% reduction for *Aedes atlanticus, Ae. Infirmatus* > 50% reduction for *An. crucians*	Qualls et al., [25]
16	USA	*Aedes albopictus*	Large-scale trial (outdoor) sprayed on plants and as ATSB trap	Industrial grade attractive sugar bait concentrate	Eugenol	0.8%	> 88% reduction with spray on plants compared with 62% reduction with trap after 4 weeks	Revay et al., [56]
17	Israel	*Aedes albopictus*	Large-scale trial (outdoor) sprayed on plants	Date syrup, citrus juice, sucrose and water	Beta- cyclodextrin microencapsulated garlic oil	0.4%	85% reduction after 26 days	Junnila et al., [57]
18	Mali	*Anopheles gambiae*	Large-scale trial (indoor)	30% guava juice, 30% honey melon juice, 25% water, 12% brown sugar and 2% local millet beer	boric acid and BaitStab™	1%	Significant reduction, 90% in female and 93% in male populations	Qualls et al. [8]
19	Israel	*Anopheles sergentii*	Large-scale trial (outdoor) sprayed on plants	Commercial attractive sugar bait concentrate	Beta-cyclodextrin encapsulated garlic-oil and blue food-dye and green food-dye	0.4%	97.5% reduction after 34 days	Revay et al., [58]
20	Jordan	*Anopheles sergentii*	Large-scale trial (outdoor)	1.5% Bacillus sphaericus and 20% sucrose	Red food- dye	1.5%	The ensuing adult population was reduced to about 60% at the experimental site	Schlein et al., [59]
21	USA	*Aedes aegypti *and *Aedes albopictus*	Small-scale trial	Mango juice, Lime juice, sugar	Boric acid and/or 1-octen-3-ol, and L-lactic acid	1%	∼ 70% reduction in *Ae. albopictus* and > 50% in *Ae. aegypti* after 72 h	Scott- Fiorenzano et al., [60]
22	USA	*Aedes albopictus*	Small-scale trial sprayed on cage plants	Mango, Lime juice and sugar solution	Boric acid and Red food-grade dye	1% and 0.5%	Applications of ATSB to leaf evaluations resulted in significant mortality of adult mosquitoes after exposed to all treated leaves, compared to untreated control leaves	Seeger et al., [31]
23	Mali	*Aedes aegypti*	Large-scale trial (outdoor) sprayed on plant	Papaya, mango, honeydew melon, leaves with extra-floral nectar	Microencapsulated garlic oil	Unknown	90–98% reduction after 36 days	Sissoko et al., [61]
24	USA	*Aedes albopictus*	Large-scale trial (outdoor)	Microencapsulated cin-namon oil-sesame oil	Yellow food dye and green food dye	0.5%	In the field, on day 11, populations at the experimental site dropped significantly	Traore et al., [35]
25	Côte d’Ivoire	*Anopheles gambiae*	Large-scale trial (indoor)	35% guava juice, 10% sugar solution	Boric acid, chlorfenapyr and orange food dye	0.2–2%, 0.05–0.5% and 2%	100% mortality with 2% boric acid and 0.5% chlorfenapyr	Furnival- Adams et al., [62]
26	Ecuador	*Aedes aegypti*	Small-scale trial (indoor)	Dried ATSB having 10% sugar	Boric acid	1%	89–91% mortality	Sippy et al., [38]
27	Mali	*Anopheles gambiae*	Large-scale trial (outdoor)	77% brown sugar and 22% date syrup	Dinotefuran and BaitStab™	0.11% and 1%	> 90% reduction in mosquito density	Traore et al., [63]
28	USA	*Aedes albopictus*	Large-scale trial (outdoor)	0.18% yeast and 88.34% sucrose	Sodium chloride	11.48%	80% mortality of male and 30% mortality of female of the target mosquito	Aryaprema, [37]
29	Mali	*Anopheles gambiae*	Large-scale trial (indoor)	~ 22% date syrup and 77% brown sugar	Orange food dye and green food dye	0.5%	16.66% and 16.19% of the females fed from fresh and aged bait stations, respectively. Of the males, 15.28% and 15.12% fed on fresh vs aged bait, respectively	Diarra et al., [64]
30	Tanzania	*Anopheles arabiensis*	Small-scale trials (indoor and outdoor)	10% brown sugar solution	Boric acid and a green food dye	2%	the ATSBs reduced outdoor-biting by 69.7% indoor biting by 79.8% and resting mosquitoes by 92.8%	Muyaga et al., [65]
31	Tanzania	*Aedes aegypti*	Small-scale trial and large-scale trial (Indoor)	10% sugar solution	Ivermectin	0.005%, 0.01%, 0.015%, 0.02%, 0.025%, 0.03%, 0.04% and 0.05%	In semi-field, ATSB reduced *Ae. aegypti* survival time compared to control-ATSB. In the field, the incidence rate ratio of captured mosquitoes increased by approximately 4.4 folds	Tenywa et al., [46]
32	West Indies and Thailand	*Aedes aegypti *and *Aedes albopictus*	Small-scale trial (outdoor)	Yeast	Benzoic acid	0.1%	100% mortality of the *Ae. albopictus* at day 7	Stewart et al., [66]
33	Taiwan	*Aedes albopictus*	Large-scale trial (outdoor)	10% sugar solution	Boric acid	1%	The total number of eggs trapped on the isolated ATSB farms was significantly lower than the total number of eggs trapped on the nonisolated ATSB farms and the control	Chiu et al., [48]
34	China	*Aedes albopictus, Culex pipiens *and *Culex tritaeniorhynchus*	Large-scale trial (outdoor)	6, 8, and 10% sugar solution	Sodium benzoate, and ammonium sulfate hydrochloride	1 g/L and 100 mg/L	After the trials, all NCBD traps effectively controlled larval and adult mosquito densities, with the highest standard decrease rates of 57.80 and 86.31%, respectively	Wu et al., [67]

Small-scale = Semi-field; Large-scale** = **real-world setting

### Context of the guidelines

ATSB, an innovative vector control tool, can curtail mosquito populations and thus reduce disease occurrence when combined with available mosquito control strategies. The Vector Control Advisory Group at World Health Organization (WHO) provides guidance to its member states on evaluation of newer vector control interventions and their associated public health value, and thus provides evidence-based recommendations for newer tools [11]. For conventional vector management methods viz., long lasting insecticide nets, residual spraying, and larvicides, the WHO has developed standard methodologies for its evaluation. These methodologies are widely accepted, leading to the approval and recommendation of these vector control tools for use in national public health programs by most countries. However, for newer tools like ATSB, there are currently no standard guidelines in place. Researchers have independently developed methodologies and have evaluated different phases of ATSB trials. For example, Kyomuhangi et al. [12] reported the Phase III evaluation of ATSB carried out in Kenya, Mali, and Zambia. Müller et al. [5] reported the field trial of ATSB for malarial mosquito control in a study conducted in the dry areas of Mali, West Africa. Qualls et al. [8] reported a proof-of-concept study to control malarial mosquitoes in Mali, West Africa using ATSB formulations.

The concept of ATSBs was first submitted to the VCAG in 2014 and in 2019 by experts of the Westham/Innovative Vector Control Consortium (IVCC). Draft protocols for three epidemiological trials of ATSBs to be carried in Africa were reviewed by the Advisory Group in May 2018 [11, 13]. As per the 18th WHO’s Vector Control Advisory Group (VCAG) report, Westham, in association with the Innovative Vector Control Consortium is reported to be working on three parallel epidemiological trials towards assessing the epidemiological effect of ATSBs on malaria programme in Kenya, Mali and Zambia [11]. The upcoming minutes of the VCAG meeting will provide more insights into the effectiveness of ATSBs in controlling vector populations, pilot programme results, Implementation challenges, and safety concerns for non-target species.

In India, the standard guidelines for evaluating vector control products against diseases of public health importance are developed by the Indian Council of Medical Research, the nodal research agency of the Health Ministry in consultation with the National Centre of Vector Borne Disease Control (NCVBDC) and regulatory authority for registration of vector control products ie., the Central Insecticide Board. These guidelines are periodically revised to incorporate the latest developments (first developed in 2005, revised in 2015, and then in 2023). Recognizing the need for newer products in vector control programme and standard methodologies for their evaluation, the ICMR has taken a futuristic approach by developing a phase-wise trial methodology for ATSB and has included it in the 3rd edition of the ICMR Common Protocol for Uniform Evaluation of Public Health Pesticides that was released in 2023 [14]. The current protocol is a structured, globally the first-ever phase-wise methodology published and in the public domain for testing ATSBs.

**Fig. 1:**
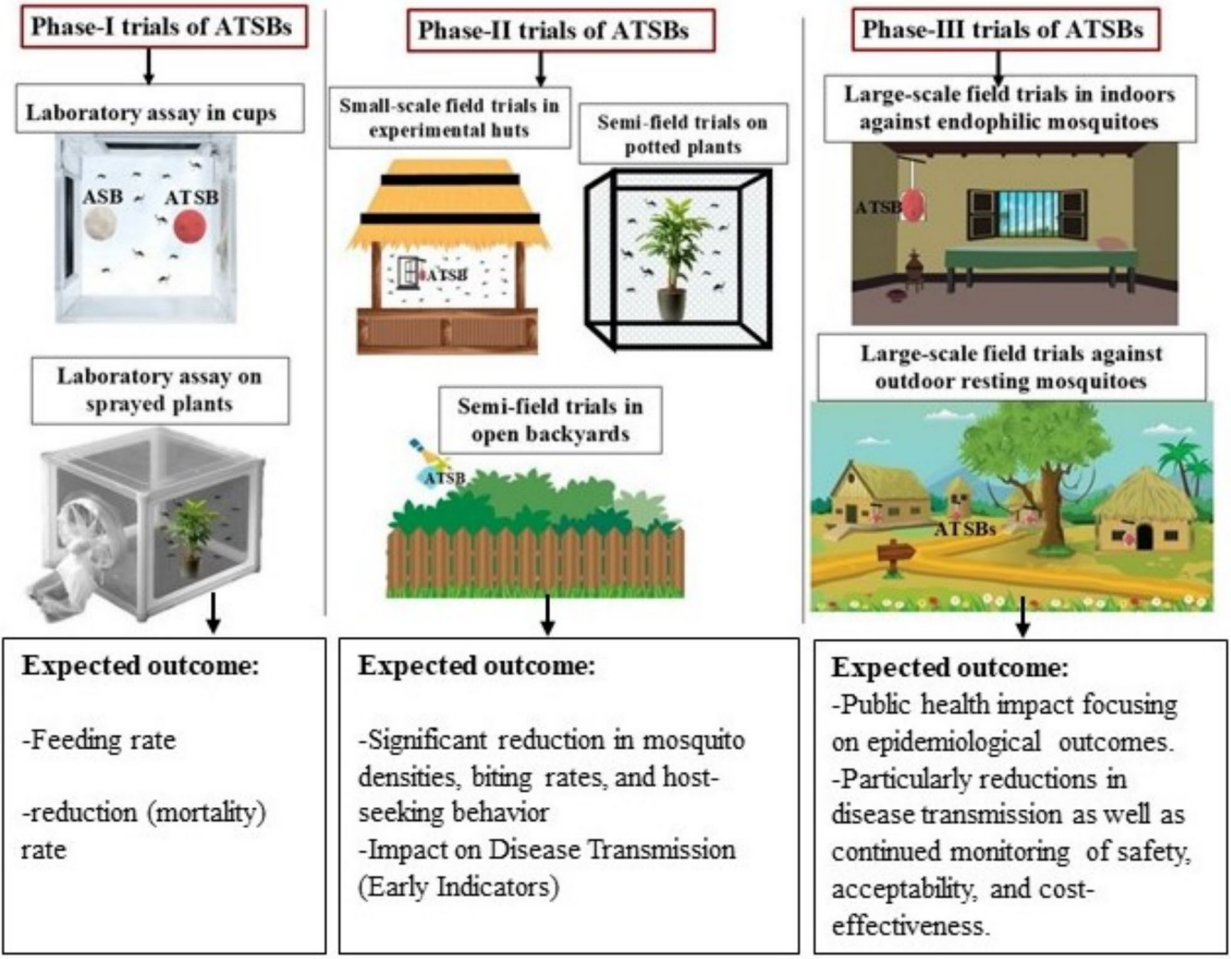
The image shows the evaluation of ATSBs in different settings

It can be utilized by researchers, industry, and regulatory bodies to conduct and review phase-wise evaluation of different formulations of ATSBs. The article outlines approaches, methods, and procedures that have been developed, tested, and refined by researchers and have been distilled from the available literature. It describes various trial settings (Fig. 1) and the types of endpoints, measurements, and observations that should be investigated and reported regarding the target vectors. Currently, there are variations across studies in attractant composition, concentration, and effective period which makes it difficult to compare efficacy. Further, inadequate testing or reporting on non-target effects in published protocols. The objective of developing the methodology is to harmonize and standardize the testing protocol while providing guidance for the evaluation of ATSBs. The methodology was prepared based on the first-hand experience with ATSB evaluation, as the authors are involved in testing new ATSB formulations. The team observed that there is no standard methodology available to be followed, and also found that variations in attractant composition, concentration, replication, and testing period in the published studies that need to be harmonized to compare the efficacy of the different ATSB formulations. The current version will be updated based on new scientific evidence and advancements in the field. In this paper, we present the phase-wise methodology in brief so as to evoke the interest of the researchers to delve deeper into the issue.

### Review of methods used in previous studies to evaluate ATSBs in the laboratory

Laboratory-based evaluations of Attractive Targeted Sugar Baits (ATSBs) are designed to assess the attractiveness of the bait formulation, to measure feeding and mortality rates in controlled conditions, to examine behavioural responses like feeding, resting, deterrence behaviour, and to compare the efficacy across mosquito species and strains (resistant vs. susceptible). The laboratory (Phase I) studies with different ATSB formulations and bait stations were analysed in detail and are summarized in Table 1. Most of the researchers used sucrose solution as attractant with varied percentages viz., Lea et al*.* [18] used 20%, some researchers used 10% [6, 7, 9, 19, 20, 24, 28, 29, 35, 39, 41, 45, 47], some used 05% [21, 25, 26, 38], others used fruit juices as attractant [22, 23, 27, 29–31, 40, 42]. In another study, fermented guava juice with 10% sucrose solution (1:1 ratio) was used as an attractant [48]. Xue and Barnard tested the effect of boric acid (1%) bait with sucrose water on mortality in *Aedes albopictus* using 25 blood-fed and 25 gravid females per cage (45 × 38 × 38 cm), and parous mosquitoes using 200 females per cage [19]. The mortality was assessed after 48 h, which was replicated four times. In the choice-based tests, they used two cages, each containing 100 male and 100 female, 5–7-day-old *Ae. albopictus* [19]. In another experiment, newly emerged 250 male and 250 female *Stegomyia albopicta* were exposed to 0.1% boric acid in 10% sucrose solution in soaked cotton balls in a cage size of 45 × 38 × 38 cm [20]. They continued the experiment till all 500 bait-exposed adults had died [20]. Allan (2011) studied the efficacy of ATSB in 10-day-old female *Culex quinquefasciatus* to bifenthrin in 10% sucrose solution, and the knockdown efficacy was evaluated at four and 24 h [21]. Similar to this, other researchers have conducted ATSB experiments with an insecticide and a varied percentage sugar solution in the laboratory against different mosquito species, but variations like the number of mosquitoes exposed, the age of the mosquitoes, the duration of the experiment, concentrations, etc., were observed [18, 22–48], which are illustrated in Table 1.

### Review of methods used in previous studies to evaluate ATSBs in small-scale and large-scale trials

Small-scale (Phase II) trials of Attractive Targeted Sugar Baits (ATSBs) are designed to assess the efficacy of ATSBs under controlled but more realistic conditions, bridging lab and full-field trials. The purpose of the large-scale (Phase III) is to evaluate the real-world effectiveness, safety, and sustainability of ATSBs under varying ecological, climatic, and entomological conditions. The small-scale and large-scale trials with different ATSB formulations and bait stations from the published sources were analysed in detail and are summarized in Table 2. Similar to laboratory study, in the phase II and phase III trials, researchers used varied percentages of sucrose solution and fruit juices as an attractant (Table 2). Muller et al*.* [15] tested Spinosad with 20% sucrose solution in two different concentrations (2% and 0.04%) by a large-scale outdoor trial sprayed on plants. In another study, Xue et al*.* [49] studied boric acid with 5% sucrose solution in 1% concentration by a small-scale outdoor trial. In another study, Muller and Schlein [50] tested Spinosad with 85% nectarines, 15% brown sugar with 02 concentrations (0.5% and 0.04%) by a large-scale outdoor trial. Similar to this, other researchers have conducted ATSB experiments with an insecticide and a varied percentage sugar solution and varied concentrations with different types of dye in the small-scale and large-scale trials against different mosquito species [4, 51–67], which are illustrated in Table 2.

### Need for a standard methodology for evaluation of ATSBs

Although numerous studies are ongoing worldwide using ATSB formulations/products, there is no uniformity observed in the testing methodologies. For this, a standard methodology needs to be developed for a uniform evaluation of ATSBs. The following methodologies which are part of the 3rd edition of Common Protocol for evaluating public health products [14] are presented for the evaluation of ATSB formulations/products under lab conditions (Phase I), simulated/small-phase (Phase II), and large-phase field trials (Phase III) for the intended use for public health by the national vector control programme.

### Preparation of ATSB and ASB formulations

The ATSB formulation can be prepared with locally available fruit juices/fruits and sugar, along with the desired concentration of the selected insecticide/toxin. The Attractive Sugar Bait (ASB) solution, which is the bait, same as above containing no active ingredient/insecticide/toxin, should be included in the testing as a control alongside the ATSB. The fruit juices can be selected based on the attractive efficacy obtained from the preliminary studies. The insecticide selection criteria for ATSB should be orally toxic to mosquitoes, safe to non-target insects, cost-effective, and insecticides that are not already used for ITNs, IRS, or larviciding to prevent resistance development. Based on the objective of the study, commercial ATSBs can also be used, as they have some advantages over homemade formulations or a known ATSB product can be used as a positive control (standard).

### Phase I trials: laboratory-based trials

#### Efficacy of ATSB formulations and bait stations on vector mosquitoes

The aim of the Phase I trial is to (a) assess the effectiveness of ATSB formulations and bait stations on vector mosquitoes and assess the continuous efficacy of ATSB formulations on plants upon spraying against mosquitoes; (b) determine the dose–response association of the ATSB formulations and bait station (6–7 concentrations) against vector mosquitoes and (c) if not known before, to find the lethal concentrations (LC) 50% and 99.9% of the ATSB and to decide its effective doses for the Phase II level test.

As a pre-requisite to the trial, a colony of the susceptible target mosquitoes need to be maintained. Use of a susceptible strain is essential, and use of F1 generation of wild mosquitoes should be in addition. Common fruit juices, preservatives, and slow-release substances can be used to prepare the formulations or bait stations. The selection will be based on the level of attraction and sugar feeding rate from the preliminary studies. The effective range of the active insecticide is to be determined by admitting the adult mosquitoes initially to 10–12 test concentrations then a narrower range of 6–7 concentrations producing mortalities amid 10–95%. Thus, LC_50_ and LC_99.9_ values can be obtained, if not known, otherwise it can be avoided. To make it easier to determine the feeding status, colour dye/coloured compound can be supplemented to the ATSB and ASB formulations or bait stations.

The efficacy of the ATSBs is determined on laboratory-reared mosquito species, 03–05 days old, non-blood-fed, 12-h-starved adults, with a male-to-female ratio of 01:01. Laboratory assays can be carried out in screened cups of 300 ml capacity or small mosquito cages of same size. Ten replicates (10 cages) with ten mosquitoes of same age in each replicate are to be used for the test arm (ATSB–6–7 concentrations) and control arm (ASB) [14] to obtain a more robust statistical analysis. After feeding on ATSB and ASB formulations for 12 h, the mosquitoes are tallied as either alive or dead, fed or unfed, the next morning. Live mosquitoes are to be introduced to new paper cups with 10% sucrose feed to observe 48–96 h delayed effect, depending upon the insecticide used. A stereo microscope or other visualisation instruments like a fluorescent microscope or UV-light torch can be used to view the dye/coloured compound through the abdomen’s cuticle will allow to identify mosquitoes that have been fed or engorged with the coloured food dye. Using newly made ATSB and ASB formulations, the test should be conducted in triplicate on different days with new batches of mosquitoes. The experiment is to *Aedes* sp. is to be carried out during the day.

#### Efficacy of ATSB formulations sprayed on plants

Target vector species raised in laboratories should be used to test ATSB containing 6–7 concentrations of an active insecticide. Every test concentration should be replicated 4–5 times in cages. The cage should be able to accommodate a locally available indoor/outdoor potted plant. Experiments are to be carried out under controlled temperature and relative humidity conditions. Flowers are removed, 50 ml of ATSB with different concentrations is sprayed covering the entire plant, and then transferred to separate study cages [14]. For the control arm, 4–5 cages containing one plant each of the same type are sprayed with ASB formulation. After an hour of spraying ATSB/ASB, 200 mosquitoes (male: female ratio 01:01), 03–05 days old and 12-h-starved are released in each cage, and mortality is noted after 48 h (72 h for slow-acting insecticides). Three iterations of the experiment are planned, each utilizing new ATSB/ASB formulations and a new group of individuals.

Assessing the ATSB’s residual toxicity in a lab setting is crucial. Similar to the experiments above, a locally available potted plant devoid of flowers is to be sprayed with 50 ml of ATSB/ASB formulations and allowed to dry. Once it is dry, 200 adults are to be introduced on day 07, 14, and 21 post-treatment and mortality is to be recorded after 48/72 h. And experiments are to be repeated thrice with fresh solutions and mosquitoes.

The insecticide (toxin) in the ATSB tested in 6–7 concentrations against the selected mosquito species can be used to determine the lethal doses (LC_50_ and LC_99.9_). The evaluation is carried out in mosquito cages with a fabric mesh screen/nylon net paced on the top fastened with rubber bands. The ATSB formulation saturated on cotton wool pads is placed on the mesh screens on the top of screening cups overnight (12 h). The effectiveness of the ATSB formulation is analysed by counting the dead mosquitoes and fed or unfed conditions after exposing the mosquitoes to the ATSB formulation. The addition of coloured food dye markers, e.g. food blue, and azorubine to the ATSB formulation enables the distinction between fed and unfed mosquitoes by examining the abdomen under a microscope [15]. After 12 h, if the mosquitoes manage to survive the exposure, the mosquitoes are transferred to separate screened cups containing sucrose feed and are then observed for delayed mortality. The lethal doses (LC_50_ and LC_99.9_) obtained in the Phase I evaluation are essential to fix the application dosages for subsequent Phase II trials.

### Phase II trials: simulated/small-scale field trials

The aim of the phase II trials is to assess the efficacy in the simulated field conditions using laboratory-reared or natural populations of targeted mosquito species. Small-scale field trials can be conducted in experimental huts using ATSB bait stations for indoor resting adults. Also, the ATSB formulation can be tested by spraying on vegetation or by using ATSB bait stations in peri-domestic outdoor localities under controlled conditions for outdoor resting vectors. However, in case of the non-availability of experimental huts, the phase II study can be conducted in the village huts with a few modest adjustments to fit the experimental hut design. The dosages of the ATSB formulation for Phase II trials are to be determined from the lethal doses obtained from the Phase I trial.

#### Evaluation of ATSBs using bait stations against mosquito vectors in experimental huts

An experimental hut can be used for each treatment (dosage) for the phase II evaluation of ATSB formulation. ATSB bait stations containing the particular concentrations of the active insecticide are placed close to the windows and suspended from the roof at the corners of the untreated bed net in each hut. Similar to how ATSB bait stations are arranged in treatment huts, ASB bait stations are also set up in the control hut. Following each trial, the knocked-out mosquitoes are gathered the next morning from the room’s floor and verandah traps for *Anopheles* and *Culex* mosquitoes. For *Aedes* mosquitoes, the knocked-out mosquitoes are gathered at the end of the experiment, during the evening hours. The dead and live mosquitoes will be collected using a mouth/mechanical aspirator. The collected mosquitoes are recognized by species and dead and bait-fed data are recorded. Live mosquitoes are to be introduced to new paper cups with 10% sucrose feed to observe 48–96 h delayed effect, depending upon the insecticide used.

#### Assessment of ATSB formulations applied to vegetation in a semi-field setting

The phase II efficacy of ATSB formulations can also be studied against laboratory-reared target mosquito species by spraying desired ATSB concentrations on potted plants under controlled screened enclosures and conditions. Post spraying, formulation on the potted plants is allowed to dry and then 3–5 days old, non-fed 500 adult mosquitoes of the target species in 01:01 sex ratio are released into each set-up and observed for mortality in treated and control after 48 h (even for 72 h for late-acting insecticides).

#### Evaluation of ATSB formulations on vegetation in open backyards

The Phase II evaluation of ATSB formulations can also be carried out by spraying on vegetation against natural vector populations during peak activity seasons to assess the effectiveness and residual activities of ATSB sprayed on plants in comparable and reasonably controlled environments. Ten residential backyards (five sites for the treatment arm and five sites for the control arm), each with a 5000–6000 m^2^ (75 × 75) area, should be selected, and the sites should be 1 km away from each. All ten sites should be comparable in terms of target mosquito density and vegetation type (Pre-evaluation analysis is to carried out). Handheld compression can be used to apply the formulation on the foliage from ground to 1 m above ground level, following a sweeping pattern (continuously from top to bottom and left to right). Traps (minimum three) can be used to monitor the efficacy of the sprayed ATSB formulation in reducing the target mosquito density on days 7, 14, 21, and can be compared with pre-treatment mosquito density data in each site and the traps should be separated by 10 m. Ovitraps can also be used to monitor the efficacy on *Aedes* mosquitoes. Separate experiments should be conducted in semi-field circumstances in regions where there aren’t enough remote, equivalent outdoor areas with comparable vegetation types where the target mosquito species are present. Three separate batches of the target vector species, as well as freshly made ATSB (treatment) and ASB (control) formulations, should be used for the efficacy investigation.

### Phase III evaluation of ATSBs against mosquito vectors at a large-scale level

The purpose of the large-scale phase III experiment is to evaluate how ATSBs affect the target mosquito species’ longevity, density, and infection/infectivity rate. In at least three distinct eco-epidemiological contexts, the prospective ATSB formulation that exhibits outstanding action in the hut-based or small-scale field trials at outdoors (Phase II) can be further assessed on a broad scale at the village level. Rain-resistant or weather-protected bait stations and ATSB formulations with water-resistant carriers, safe for humans and animals, are recommended for phase-III trials. Thus, the main outcome of the phase III trial will be the effectiveness of ATSBs in real-world conditions, safety, and sustainability of ATSBs under varying ecological, climatic, and entomological conditions.

#### Selection of study villages for phase III evaluation of ATSB formulations and bait stations

Villages are the unit of investigation in the cluster-randomized phase III trials. Six villages are to be selected based on the density of the target vector species and similar vegetation with an approximate human population of 1000 people. A minimum distance of 2 km is maintained to prevent mosquito permeation between the treatment and control villages.

For ATSB treatment, three villages (3 treatment arms) with an approximate population of about 1000 should be chosen. For comparing the results, three villages with similar populations ought to be selected as a control village (3 control arms). Prior to treatment, using suitable sample techniques, the target vector species’ density should be checked for a month before commencing the spraying, considering the mosquito peak seasons in each of the six villages that were chosen. The ATSB (treatment) and ASB (control) formulations, along with the construction of bait stations, can be made using the same components as those utilized in the phase II trial.

#### Phase III evaluation of ATSB bait stations laid indoors against endophilic mosquitoes

In the chosen experimental study villages, ATSB bait stations are installed in each room (01/room) during the transmission season after the pre-treatment assessment. By choosing at least 10 homes at random each sample period, the target mosquito species must be observed every week or every 2 weeks in both the treatment and control areas, depending on the residual activity of the product in order to predict the impact of the ATSB bait stations at indoors. Pyrethrum spray catches (PSC) or hand catch techniques are used to sample every room in the ten households that have been assigned. According to the results of the Phase II trial, all of the bait stations should also be changed out periodically for fresh ones when the mortality falls significantly < 100%, or typically < 80%. All the collected mosquitoes are then identified to species, and the male–female ratio, their sugar feeding status, blood-feeding status, and parity status can be studied. The primary outcome measure will be the percent reduction in indoor adult female mosquito density per house per night in intervention vs. control arms, measured by standardized sampling methods and epidemiological outcomes. The effect size can be set as a 40–50% reduction in mosquito density, and the entomological sample size formula can be used to determine the entomological outcome.

#### Phase III evaluation of ATSB formulations against outdoor resting mosquitoes

This study purposes to assess the effectiveness of the ATSB formulations against outdoor resting mosquito vectors by spraying on vegetation. The trial is carried out outdoors, preferably in residential backyards during the respective mosquito transmission seasons. At least six identical outdoor locations, each with a population of roughly 1000 people, can be chosen for this outdoor study based on visually apparent similarities. The target vector species’ larval habitats should be sufficiently numerous at these chosen locations, ideally with similar densities of target mosquito species. To prevent mosquitoes from infiltrating the treatment region, the control locations should be chosen at least 2 kms distance from the chosen study sites, while the test sites may be assigned at random. Based on the sufficient density of target vector mosquitoes gathered during pre-trial surveys, the study sites ought to be chosen. The vegetation in residential backyards is sprayed with the ideal dosage of the ATSB formulation, which was established in the phase II study. The post-treatment mosquito density is monitored for 1–7 days for detecting early reductions of ATSB-fed mosquitoes, 2–4 weeks for detecting significant density reduction, 5–8 weeks to assess sustained impact, and up to 12 weeks (3 months) for persistence evaluation of bait attractiveness and residual effect following the same methods and traps as used in the pre-density survey. If there is rain, reapply the ATSB following the original application method and restart the trial. It is important to assess the environmental conditions (humidity and rain forecast) before starting each ATSB spray application. If possible, ATSB formulations with water-resistant carriers can be chosen.

Depending on their residual activity, the ATSB and ASB bait stations should be changed every week or every 2 weeks. Pre- and post-treatment collections should be compared, and their processing should involve identifying the mosquitoes, determining their percent parity, and determining whether they are blood-fed. Further, the vector infection rate should be assessed to determine vector infection/infectivity rates by examining the midgut of female mosquitoes under the microscope [16] and salivary glands using ELISA-based or PCR-based methods [17]. If the ATSB study is carried out on malarial vectors, then the entomological inoculation rate (EIR) can be calculated to assess the total infective bites per individual per night.

### Toxicity and environmental safety

The toxicant in the ATSB formulation is typically a chemical insecticide that is included to kill vector mosquitoes, but it may be a threat to other organisms, including beneficial insects, and mammals. Ideally, ATSBs are formulated to be species-specific, targeting only vector mosquitoes. However, non-target species may also ingest the ATSB toxicant. For example, beneficial insects like honeybees may be attracted to the sugar used in the ATSB and unintentionally consume the bait. This can cause unintended effects on other organisms if the ATSB toxicants are not deployed appropriately. While ATSBs are an effective vector control tool, particularly for mosquito control, their toxicity and environmental safety must be carefully considered. The risks to non-target organisms and the environment can be reduced through careful selection of biodegradable insecticides, appropriate use, and proper deployment.

## Conclusion

The methodology/testing framework will facilitate researchers in consistently generating data across various studies and settings. This will enable comparisons of the results of different ATSB formulations/products carried out by different organizations. It will also aid in assessing the suitability of ATSB-based products for controlling vector mosquitoes in different ecotypes.

## Data Availability

No datasets were generated or analysed during the current study.

## Abbreviations

ATSB: Attractive Targeted Sugar Bait
ASB: Attractive sugar bait
IVM: Integrated vector management
VBDs: Vector borne diseases
UV-light: Ultraviolet light
F1: First generation
LC: Lethal concentrations
ELISA: Enzyme-linked immunosorbent assay
PCR: Polymerase chain reaction
PSC: Pyrethrum spray catches
ITNs: Insecticide-treated nets
IRS: Indoor residual spray
ICMR: Indian council of medical research
NCVBDC: National centre of vector borne diseases control
IVCC: Innovative vector control consortium
WHO: World Health Organization
